# Homotopic Connectivity in Early Pontine Infarction Predicts Late Motor Recovery

**DOI:** 10.3389/fneur.2018.00907

**Published:** 2018-10-31

**Authors:** Yi Shan, Yin-Shan Wang, Miao Zhang, Dong-Dong Rong, Zhi-Lian Zhao, Yan-Xiang Cao, Pei-Pei Wang, Zheng-Zheng Deng, Qing-Feng Ma, Kun-Cheng Li, Xi-Nian Zuo, Jie Lu

**Affiliations:** ^1^Department of Radiology, Xuanwu Hospital, Capital Medical University, Beijing, China; ^2^Key Laboratory of Magnetic Resonance Imaging and Brain Informatics, Beijing, China; ^3^Department of Psychology, University of Chinese Academy of Sciences, Beijing, China; ^4^Key Laboratory of Behavioral Science, Magnetic Resonance Imaging Research Center, Institute of Psychology, Chinese Academy of Sciences, Beijing, China; ^5^Department of Neurology, Xuanwu Hospital, Capital Medical University, Beijing, China; ^6^Department of Nuclear Medicine, Xuanwu Hospital, Capital Medical University, Beijing, China

**Keywords:** pontine infarction, homotopic connectivity, functional magnetic resonance imaging, motor recovery, early prediction

## Abstract

Connectivity-based methods are essential to explore brain reorganization after a stroke and to provide meaningful predictors for late motor recovery. We aim to investigate the homotopic connectivity alterations during a 180-day follow-up of patients with pontine infarction to find an early biomarker for late motor recovery prediction. In our study, resting-state functional MRI was performed in 15 patients (11 males, 4 females, age: 57.87 ± 6.50) with unilateral pontine infarction and impaired motor function during a period of 6 months (7, 14, 30, 90, and 180 days after stroke onset). Clinical neurological assessments were performed using the Fugl–Meyer scale (FM).15 matched healthy volunteers were also recruited. Whole-brain functional homotopy in each individual scan was measured by voxel-mirrored homotopic connectivity (VMHC) values. Group-level analysis was performed between stroke patients and normal controls. A Pearson correlation was performed to evaluate correlations between early VMHC and the subsequent 4 visits for behavioral measures during day 14 to day 180. We found in early stroke (within 7 days after onset), decreased VMHC was detected in the bilateral precentral and postcentral gyrus and precuneus/posterior cingulate cortex (PCC), while increased VMHC was found in the hippocampus/amygdala and frontal pole (*P* < 0.01). During follow-up, VMHC in the precentral and postcentral gyrus increased to the normal level from day 90, while VMHC in the precuneus/PCC presented decreased intensity during all time points (*P* < 0.05). The hippocampus/amygdala and frontal pole presented a higher level of VMHC during all time points (*P* < 0.05). Negative correlation was found between early VMHC in the hippocampus/amygdala with FM on day 14 (*r* = −0.59, *p* = 0.021), day 30 (*r* = −0.643, *p* = 0.01), day 90 (*r* = −0.693, *p* = 0.004), and day 180 (*r* = −0.668, *p* = 0.007). Furthermore, early VMHC in the frontal pole was negatively correlated with FM scores on day 30 (*r* = −0.662, *p* = 0.013), day 90 (*r* = −0.606, *p* = 0.017), and day 180 (*r* = −0.552, *p* = 0.033). Our study demonstrated the potential utility of early homotopic connectivity for prediction of late motor recovery in pontine infarction.

## Introduction

Impairment of motor function after ischaemic stroke remains a leading cause of long-term adult disability, with an incidence of up to 2 in 3 stroke survivors (1). Assessment of brain integrity and time-dependent reorganization in multiple cortical networks play a decisive role in better outcomes of motor recovery after stroke (2, 3). In clinical practice, it is extremely valuable to identify feasible bio-markers in patients with early stroke to maximize the possibility of predicting late behavioral recovery. At present, resting-state functional magnetic resonance imaging (rs-fMRI) is one of the most recognized non-invasive techniques revealing cortical alterations in neurological or psychiatric patients. Functional connectivity (FC) obtained by rs-fMRI could observe spontaneous neuronal activities by calculating temporal consistency in low frequency oscillation of blood oxygenation level dependent (BOLD) signals between any pair of spatially remote brain regions in the whole brain (4, 5). Rs-fMRI is especially appropriate for patients with stroke because of several of the following reasons: (a) it is easy to operate with patients who are severely motor impaired; (b) we can study multiple functional networks with a single scan; (c) it is available at both group-level and single-level analysis; (d) abnormal alterations of rs-fMRI could be detected within hours after stroke onset, which makes it possible to reveal the neuronal mechanism underlying brain reorganization from a very early stage (6–9). Therefore, this connectivity-based method is essential to explore brain reorganization after stroke and to provide meaningful predictors for late motor recovery.

Previous studies have discovered that disruptions of inter-hemispheric FC between homologous brain areas in patients with early stroke correlates with their behavioral performances indicating motor dysfunction (10). Decreased inter-hemispheric FC in the somatomotor resting-state network was significantly correlated with clinical assessment in patients with acute stroke (11). In chronic stroke, connectivity between homotopic FC was stronger than that in intra-hemispheric FC and was significantly associated with clinical performance of upper-extremity control (12). Thus, homotopic FC changes seem to be adequate biomarkers for evaluation of post-stroke motor recovery. However, several problems from these studies need to be further discussed: (a) As well as the sensorimotor network, focal stroke lesions could also affect multiple other networks, such as the attention and cognitive network (13–17). Therefore, whole-brain homotopic FC should be taken into account. (b) The lack of longitudinal observation limits most cross-sectional studies when characterizing time frames and trajectories of long-term homotopic FC changes during motor recovery. (c) Concerning the inclusion criteria of most of the above studies, the location of individual stroke lesions was variable in the patient group. However, location–dependent research is essential in understanding different patterns of post-stroke brain reorganization because lesion localization in early stroke has been shown to affect patients' functional outcome (18). On the other hand, lesions that directly damage focal cortical structure or indirectly influence the haemodynamic status of involved brain cortex will both influence BOLD signals of RSNs. Therefore, it would be of innovative significance to conduct a longitudinal rs-fMRI study on characteristic patients with stroke who have a simple subcortical lesion localization.

To address the aforementioned problems, we aim to investigate the whole-brain homotopic FC alterations during a 180-day follow-up of patients with pontine infarction. Pontine infarction is one of the most common types of cerebral stroke leading to motor dysfunction. Unlike other subcortical lesions normally reported in previous studies (mostly located in the internal capsule and neighboring regions), the pontine region is a subtentorial structure remote from the cerebral cortex. A recent study has demonstrated different patterns of whole-brain structural damage in patients with pontine stroke and capsular stroke (19). Therefore, we suppose patients with pontine infarction would present different patterns of functional impairment compared with other infarcts. Thus, we hypothesize that characteristic time frames and trajectories of homotopic FC alterations would be presented in patients with pontine infarction, compared with healthy controls. We also predict that early changes of inter-hemispheric FC in patients with pontine infarction would be related to dynamic behavior assessment of motor function during the 180-day follow-up, hence early homotopic FC would be a prospective biomarker for late motor recovery prediction.

## Materials and methods

### Participants

**Figure 1:**
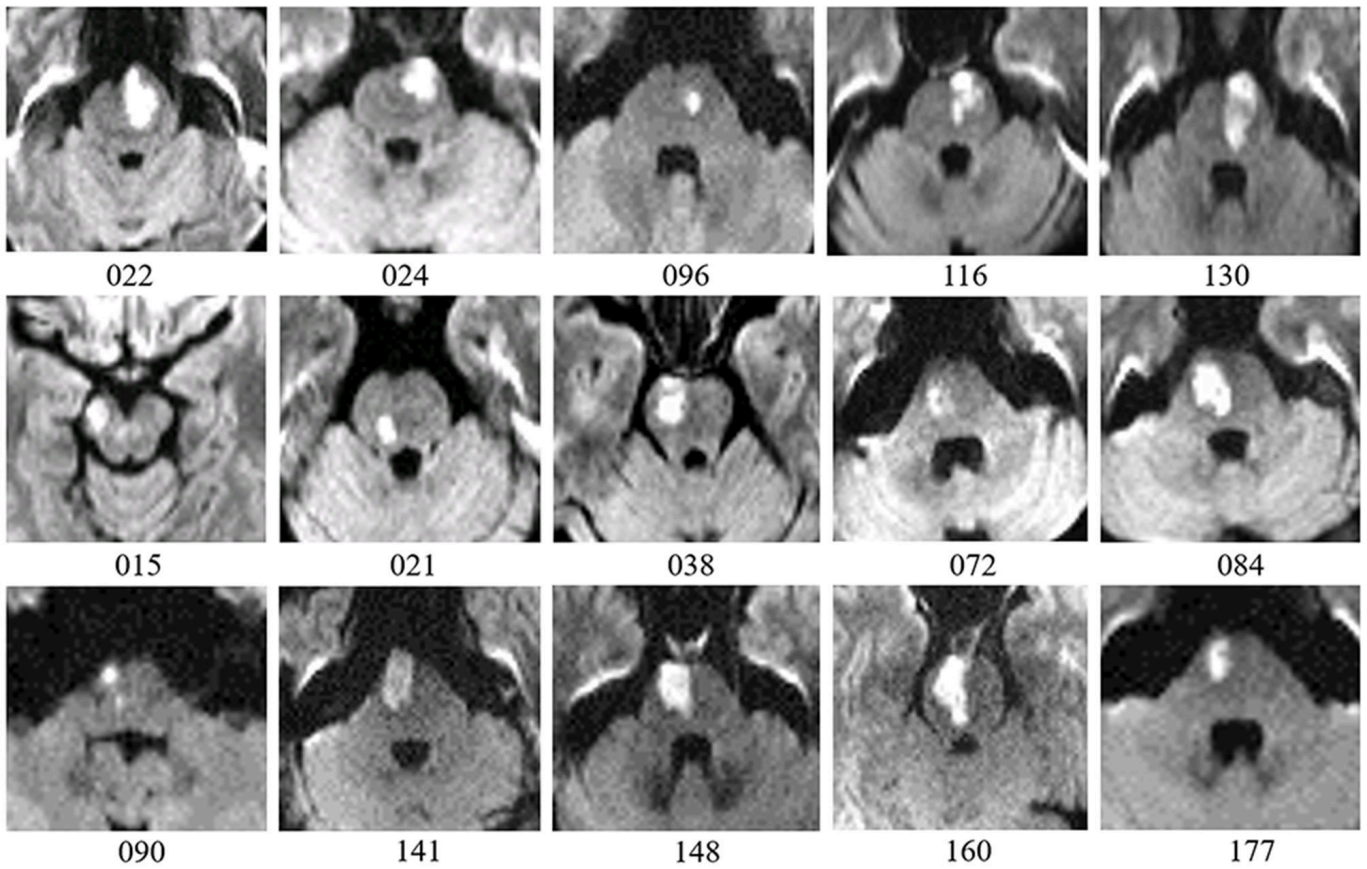
Lesion details of patients with pontine infarction are depicted in axial DWI maps. Number below each image is the participant ID presented in Table 1. Five patients with lesions on the left side are placed on the first row, while 10 patients with lesions on the right side are shown on the following 2 rows. For all images, right is displayed on the left (radiological convention).

All patients with pontine infarction were recruited from the Department of Neurology, Xuanwu Hospital, Capital Medical University (Beijing, China), given informed consent and details of study procedures. Our study was approved by the Medical Research Ethics Committee of Xuanwu Hospital. Inclusion criteria were as follows: (a) patients were enrolled within 7 days of the onset of symptoms; (b) a single pontine infarction with a unilateral lesion was identified on MRI; (c) there were mild to severe motor deficits of contra-lesional extremities; (d) there was a detailed history of the hospitalization period and clinically stable medical status during follow-up. The exclusion criteria were as follows: (a) any contraindication to MRI; (b) any other brain abnormalities detected with MRI; (c) any history of neurological diseases or psychiatric disorders; (d) any recurrence of stroke or secondary hemorrhage during follow-up. Finally, a total of 15 patients with pontine infarction (11 males and 4 females, 58.1 ± 6.4 years) completed 5 visits over a period of 6 months. According to the diffusion-weighted imaging maps presented from the initial MRI scan, 10 pontine infarction lesions took place on the right side, while 5 other lesions occurred on the left side. The volumes of these lesions ranged from 2.4 mL to 23.2 mL (11.4 ± 6.6 mL). See Figure 1 for more details of the infarct lesions. Apart from the symptoms of hemiparesis, 6 of the patients had dizziness and 5 of the patients had dysarthria as well. See Table 1 for more demographic data and clinical characteristics of the patients. We also recruited 15 right-handed healthy controls (11 males and 4 females, 56.1 ± 6.4 years) matched to the patients in age and gender, all the brain imaging data of these healthy controls have been publicly shared via the Consortium for Reliability and Reproducibility (CoRR) (20). They were volunteers without any neurological disease or abnormal findings on MRI images. All subjects were right-handed, measured with the Edinburgh Handedness Inventory (21).

**Table 1:** Demographics, clinical characteristics, and motor function scores of patients with pontine infarction.

**Participant ID**	**Age (years)**	**Gender**	**Lesion side**	**Symptoms**	**FM**				
					**<7days**	**14 days**	**30 days**	**90 days**	**180 days**
015	54	F	R	Hemiparesis	75.0	90.9	95.5	98.5	100.0
021	57	M	R	Hemiparesis	88.6	94.7	96.2	100.0	99.2
022	59	M	L	Hemiparesis, dysarthria	66.7	81.8	86.4	100.0	97.0
024	48	M	L	Hemiparesis, dysarthria	50.0	68.2	75.8	74.2	78.0
038	63	M	R	Hemiparesis	62.9	83.3	96.2	100.0	100.0
072	61	M	R	Hemiparesis, dysarthria	75.0	90.9	99.2	98.5	97.0
084	56	M	R	Hemiparesis	44.7	14.4	40.2	43.2	57.5
090	68	M	R	Hemiparesis, dizziness	100.0	100.0	100.0	100.0	100.0
096	62	M	L	Hemiparesis, dizziness	72.7	90.2	98.5	90.9	98.5
116	62	F	L	Hemiparesis, dizziness	45.5	72.7	81.1	77.3	85.6
130	60	M	L	Hemiparesis, dysarthria, dizziness	/	34.9	69.7	83.3	95.5
141	56	M	R	Hemiparesis	27.3	47.0	89.4	98.5	100.0
148	64	F	R	Hemiparesis, dizziness	6.1	7.6	40.9	60.6	74.2
160	42	M	R	Hemiparesis, dizziness	15.2	28.8	47.0	69.7	83.3
177	56	F	R	Hemiparesis, dysarthria	/	50.0	80.3	90.9	100.0

*F, female; M male; L, left; R, right; FM, Fugl–Meyer score*.

### MRI data acquisition

Patients with pontine infarction underwent the first session of MRI scanning within 7 days after stroke onset and on 4 subsequent sessions during each visit (14, 30, 90, and 180 days after stroke onset). The healthy control group also underwent 5 sessions of MRI (14, 30, 90, and 180 days after the first visit). All data were obtained with a 3T scanner (MAGNETOM Tim Trio, Siemens Healthcare, Erlangen, Germany) using the 12-channel phased-array head coil. During the scanning procedure, participants were instructed to keep their eyes open and stay awake, without any other provided task. Structural images were acquired with a sagittal MP-RAGE three-dimensional T1-weighted sequence (repetition time = 1,600 ms, echo time = 2.15 ms, flip angle = 9°, voxel sizes = 1.0 × 1.0 × 1.0 mm^3^, field of view = 256 × 256). Functional images, with 124 whole-brain images at each session, were acquired using the gradient-echo echo-planar pulse sequence (repetition time = 3,000 ms, echo time = 30 ms, flip angle = 90°, number of slices = 43, voxel sizes = 3.0 × 3.0 × 3.0 mm^3^, matrix size = 64 × 64).

### Behavioral assessments

**Figure 2:**
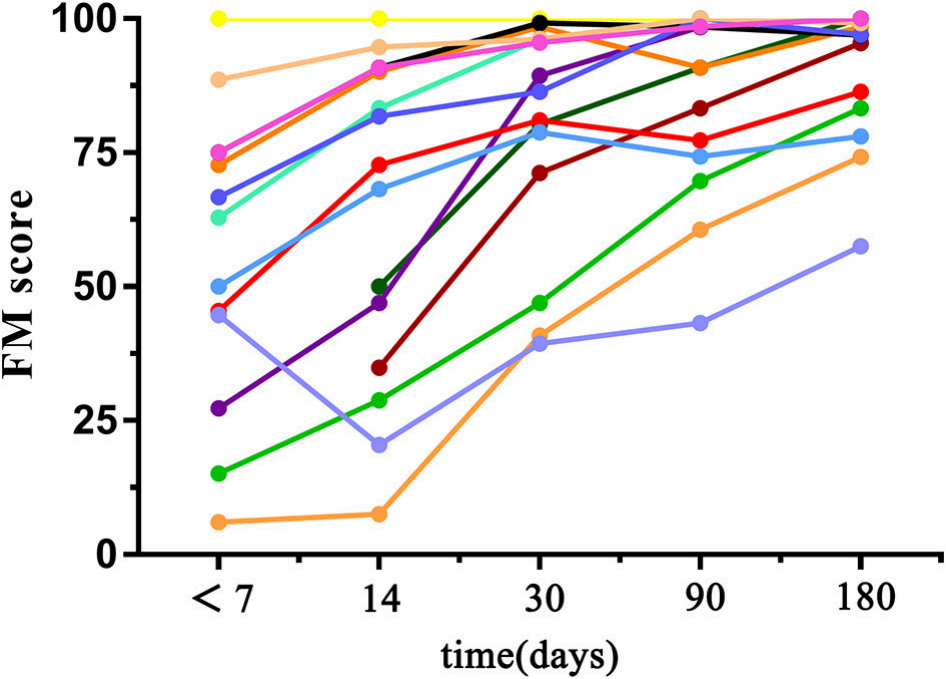
Graph shows changes in FM scores with time in patients. Each line indicates the trend of behavior recovery of one patient.

Clinical assessments including motor function, balance, sensation, and joint function of the upper limbs were evaluated by using 33 related tasks in the Fugl–Meyer (FM) Assessment. Each score was given on a scale of 0 to 2, according to the patient's response to the specific task (0, patient was unable to perform the task, 1, patient could partially perform the task, 2, patient could accomplish the task). All cores of the total 33 tasks were summed and normalized to a score between 0 and 100. During each visit, 2 neurologists (QM and a non-author with over 10 years of experience in clinical practice) independently performed behavioral assessments before and after MRI scanning (the scores assessed by the two raters were significantly correlated; *r* = 0.998, *p* < 0.001). Afterwards, these 2 scores were averaged to reduce interrater differences. All patients presented good motor recovery to some extent during the 6 months of follow-up, with FM scores gradually increased (52.11 ± 30.83, 63.69 ± 30.91, 79.75 ± 21.28, 85.71 ± 17.45, 91.06 ± 12.75 within 7 days, 14, 30, 90, and 180 days after stroke onset, respectively). FM scores between follow-up visits were significantly correlated with baseline scores (day 14, *p* = 0.000, *r* = 0.906; day 30, *p* = 0.001, *r* = 0.786; day 90, *p* = 0.016, *r* = 0.649; day 180, *p* = 0.039, *r* = 0.576). During the last visit, 10 patients were able to return to work, 2 were completely dependent and 3 were partially dependent. See Table 1 and Figure 2 for detailed FM at each visit and the trends of behavioral recovery of each patient.

### Data analysis

Both structural and functional image pre-processing were conducted with the Connectome Computation System (CCS: http://lfcd.psych.ac.cn/ccs.html), which provides a common platform for brain connectome analysis by integrating the functionality of AFNI, FSL, and FREESURFER software. A concise description of CCS steps can be found in Zuo et al. (22). Briefly, the structural-processing steps included the process of removal of noise, brain surface reconstruction, spatial normalization and boundary-based registration. Functional pre-processing pipeline included discard of the first 5 EPI volumes (10 s), removal and interpolation of temporal spikes, slice-timing correction, motion correction, 4-D global normalization to mean intensity 10,000, boundary-based registration to match individual functional images to structural images, motion correction with Fristion's 24-parameter regression, correction of WM and CSF mean time series of individual rfMRI time series, band-pass filter (0.01–0.1 Hz), removal of linear and quadratic trends as well as Gaussian spatial smoothing (FWHM = 6 mm).The data were then projected onto a symmetric fsLR32k surface grid template. Finally, we flipped images of the patients with a left infarcted lesion horizontally to the right side.

Whole-brain homotopic connectivity was defined as the resting state functional connectivity between each pair of symmetric interhemispheric vertex, with a method called voxel-mirrored homotopic connectivity (VMHC) (23). For every subject, we calculated Pearson's correlation coefficient between each vertex's residual time series and that of its corresponding interhemispheric part. A detailed processing method has been described in Zuo et al. (23). Vertex -wise 2-sample *t*-tests were performed to find significant differences on VMHC maps between patients and healthy controls. Additionally, Z-statistic maps of clusters were defined by a threshold of *Z* = 2.3 and a cluster-level threshold based on Gaussian Random Field theory with a cluster-level family-wise error (FWE) correction for multiple comparisons (corrected *p* < 0.05).

First of all, we compared VMHC differences between patients and healthy controls at the first time point (<7 days after stroke onset) to find out early homotopic connectivity changes in acute pontine infarction. Afterwards, for patients, we set the clusters with significant differences on the first time point as the region of interest (ROI), and extracted dynamic alterations of their VMHC during the 6-month recovery period (visits on day 14, 30, 90, and 180 after stroke onset). For healthy controls, we averaged the mean VMHC value of these ROIs at all 5 visits. Then we analyzed the time-dependent differences of VMHC value in patients on day 14, day 30, day 90, and day 180 compared with the average of healthy controls over 5 time points, by paired 2-sample *t*-tests, to show the dynamic alternations of these brain areas. When analyzing how early homotopic connectivity changes predicts late motor recovery in patients, we used Pearson correlation to evaluate correlations between VMHC value at the first time point (<7 days after stroke onset) and the behavioral measures on subsequent 4 visits, respectively (visits on day 14, 30, 90, and 180 after stroke onset). A significant difference indicated by *P*-value of <0.05 (2-tailed) for all statistical procedures.

## Results

### Homotopic connectivity differences between patients with early pontine infarction and healthy controls

**Figure 3:**
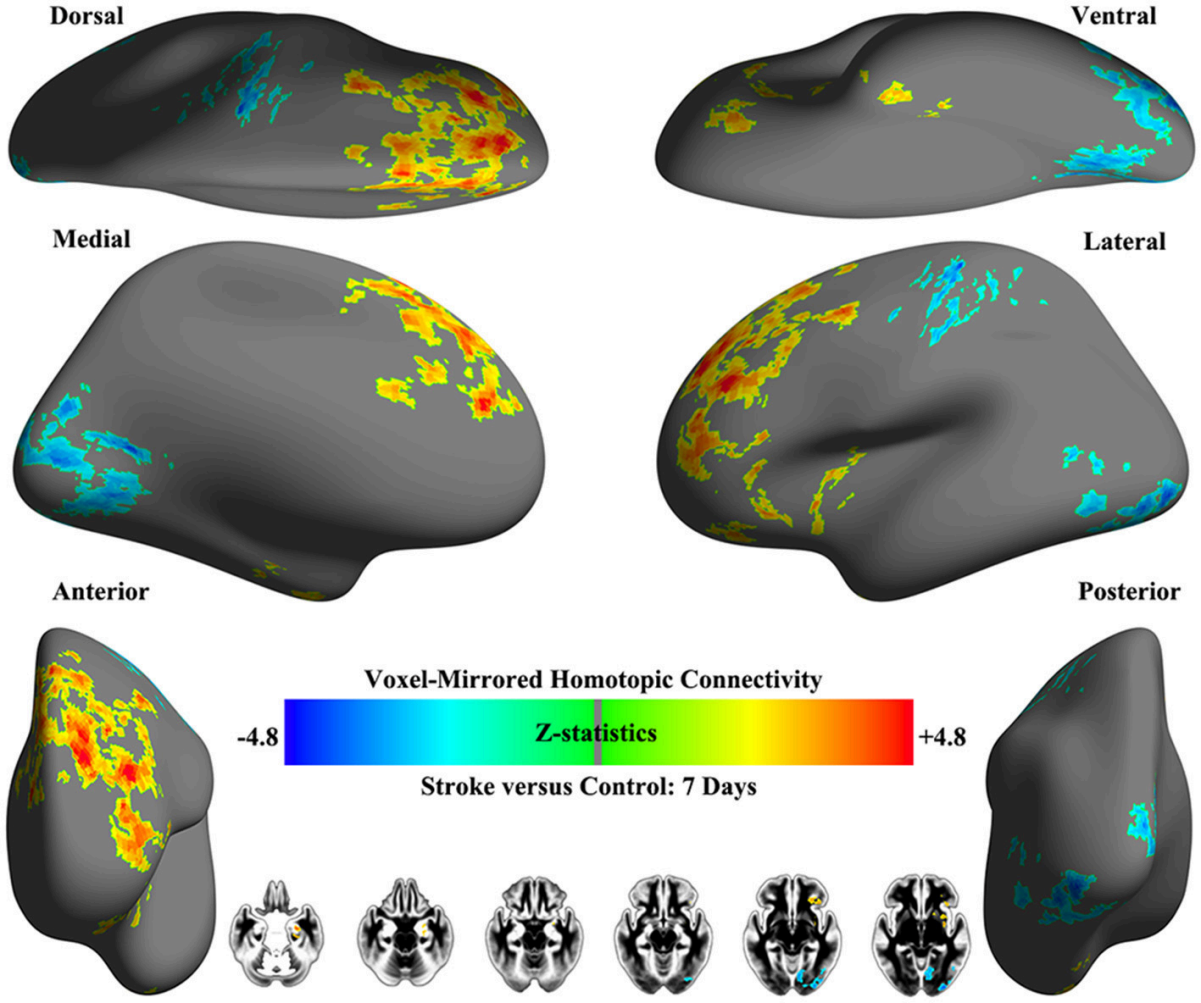
Whole-brain surface renderings of group differences in VMHC between patients (within 7 days after stroke onset) and healthy controls. Z-statistic maps (Minimum *Z* > 2.3; cluster level, *p* = 0.05, corrected) are pictured as 6 hemispheric surfaces (cortical regions) and 6 symmetric axial slices (subcortical regions). Warm color indicates brain areas with increased VMHC in patients with stroke while cold color indicates brain areas with decreased VMHC compared with healthy controls.

Obvious differences of VMHC intensity were detected between patients with early pontine infarction (within 7 days after onset) and healthy controls in 4 pairs of brain areas on visual inspection, including decreased VMHC values in the bilateral precentral and postcentral gyrus and precuneus/posterior cingulate cortex (PCC), as well as increased VMHC in the hippocampus/amygdala and frontal pole (*P* < 0.01). The VMHC changes with the biggest cluster size were presented in the frontal pole, followed by the precuneus/PCC, hippocampus/amygdala and precentral and postcentral gyrus. See Table 2 and Figure 3 for details.

**Table 2:** Brain areas showing differences in VMHC between patients with pontine infarction and healthy controls.

**Brain area**	**VMHC changes**	**Voxels**	***Z*-value^a^**	**Talairach coordinates**		
				**X**	**Y**	**Z**
Post/pre central gyrus	Decrease	564	−4.22	59	48	67
Precuneus/PCC	Decrease	1835	−4.72	54	32	41
Hippocampus/amygdala	Increase	734	4.53	59	59	23
Frontal pole	Increase	3554	5.25	56	85	53

*VMHC, voxel-mirrored homotopic connectivity; PCC, posterior cingulate cortex*.

a *Maximum z-statistic*.

### Dynamic changes of homotopic connectivity in patients with pontine infarction during the 6-month recovery period

**Figure 4:**
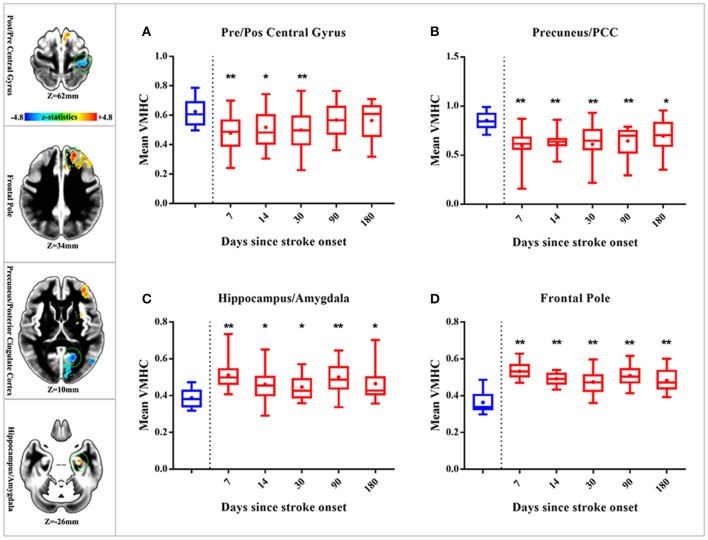
Dynamic changes of whole-brain VMHC in patients with stroke compared with healthy controls. The 4 axial slices on the left vertical row show the position of brain areas (marked with a green circle) with significant VMHC differences in patients with early stroke (located in the precentral and postcentral gyrus, precuneus/PCC, hippocampus/amygdala and frontal pole, respectively). Warm color indicates increased VMHC, while cold color indicates decreased VMHC. The 4 box plots on the right show time-dependent changes in 4 brain regions [precentral and postcentral gyrus **(A)** precuneus/PCC **(B)** hippocampus/amygdala **(C)** frontal pole **(D)**] over 5 time points (within 7 days, 14 days, 30 days, 90 days, and 180 days after stroke onset). Red boxes indicate the mean VMHC of patients with stroke, while blue boxes indicate that of healthy controls. “**” represents significant differences between patients and HC at a threshold of *P* < 0.01; “*” represents a threshold of *P* < 0.05. VMHC, voxel-mirrored homotopic connectivity; HC, healthy controls; PCC, posterior cingulate cortex.

Here, we further observed time-dependent changes of VMHC in the 4 brain areas above (precentral and postcentral gyrus, precuneus/PCC, hippocampus/amygdala, and frontal pole) which presented decreased or increased VMHC differences in patients with early pontine infarction. See Figure 4 for more details. The VMHC levels in the precentral and postcentral gyrus showed decreased intensity in patients with pontine infarction compared with that of healthy controls on day 14 (*P* < 0.05) and day 30 (*P* < 0.01), but increased gradually to the normal level from day 90. However, VMHC in the precuneus/PCC presented decreased intensity during all 4 visits; these values were significantly different on day 14 (*P* < 0.01), day 30 (*P* < 0.01), day 90 (*P* < 0.01), and day 180 (*P* < 0.05). For brain areas that showed increased VMHC in patients with early pontine infarction, the hippocampus/amygdala and frontal pole showed a persistently higher level of intensity than that in healthy controls at all time points. Significant differences were observed on day 14 (*P* < 0.05), day 30 (*P* < 0.05), day 90 (*P* < 0.01), and day 180 (*P* < 0.05) in the hippocampus/amygdala, while the values for the frontal pole showed a significant difference (*P* < 0.01) between patients and healthy controls at all 4 time points.

### Correlations between homotopic connectivity changes in early pontine infarction and late motor recovery

**Figure 5:**
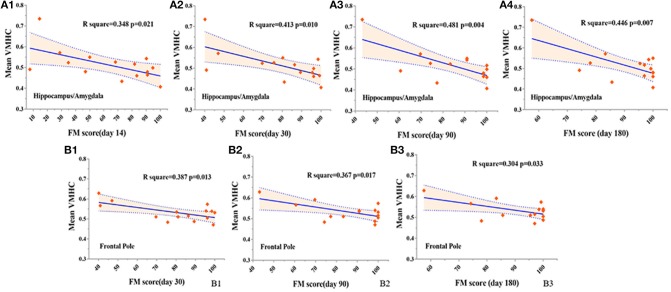
Correlations between VMHC changes (within 7 days after stroke onset) and FM (4 visits during 6 months) in patients with stroke. In the hippocampus/amygdala, negative correlations are shown between VMHC and FM on day 14 **(A1)**, day 30 **(A2)**, day 90 **(A3)**, and day 180 **(A4)** after stroke onset, at a threshold of *P* < 0.05. In the frontal pole, negative correlations are presented between VMHC and FM on day 30 **(B1)**, day 90 **(B2)**, and day 180 **(B3)** after stroke, at a threshold of *P* < 0.05. VMHC, voxel-mirrored homotopic connectivity. FM, Fugl–Meyer score.

When we correlated decreased homotopic FC in early pontine infarction (in the precentral and postcentral gyrus and precuneus/PCC) with behavioral performance on 4 visits (14, 30, 90, and 180 days after stroke onset), we did not find any relationship between VMHC value and FM scores which were significantly different. However, regions with increased VMHC within the 7 days after stroke (in the hippocampus/amygdala and frontal pole) showed a significant correlation with late motor recovery. VMHC values in the hippocampus/amygdala were negatively correlated with FM scores on day 14 (*r* = −0.59, *p* = 0.021), day 30 (*r* = −0.643, *p* = 0.01), day 90 (*r* = −0.693, *p* = 0.004) and day 180 (*r* = −0.668, *p* = 0.007). Furthermore, VMHC in the frontal pole was negatively correlated with FM scores on day 30 (*r* = −0.662, *p* = 0.013), day 90 (*r* = −0.606, *p* = 0.017), and day 180 (*r* = −0.552, *p* = 0.033). See Figure 5 for more details.

## Discussion

We investigated overall homotopic FC alterations in early pontine infarction, the dynamic pattern of brain reorganization during a 180-day follow-up and the relationship between early VMHC changes and late motor recovery in these patients. Our observations showed 4 pairs of brain areas with characteristic time-dependent VMHC changes in patients with pontine infarction. The two regions with reduced VMHC in early stroke showed a tendency to increase during follow-up: VMHC in the precentral and postcentral gyrus rose to normal levels from day 90, while VMHC in the precuneus/PCC showed a continuously lower level in comparison with healthy controls until day 180. The two regions with increased VMHC in early stroke did not appear to change during follow-up: the VMHC values in the hippocampus/amygdala and frontal pole showed a persistently higher level than in the healthy controls during the recovery period. In addition, brain areas with increased VMHC in early pontine infarction showed significant negative correlation with late motor assessment. Thus, we consider that homotopic FC changes in the hippocampus/amygdala and frontal pole may be prospective indications for motor recovery in these patients.

### Dynamic functional homotopy across the recovery period in patients with pontine infarction

In our work, disrupted functional homotopy was initially detected in the precentral and postcentral gyrus and precuneus/PCC, which are important components in the sensorimotor network and default mode network, respectively. In previous studies, decreased homotopic FC in sensorimotor area could be detected from within 24 h to 1–12 weeks after stroke onset, and it increased to different levels (normal, higher or still lower than normal people) in the final stable stage (90 days to 1 year). The changes in FC showed large differences in time-dependent trajectories (8, 12, 24–28). These dynamic enhancements of FC are commonly considered to play beneficial roles in motor restoration. In our findings, homotopic FC in the precentral and postcentral gyrus quickly increased to normal levels on day 90, which may indicate the characteristic pattern of functional reorganization after pontine infarction or be related to a comparatively rapid motor-recovery process. On the other hand, initially abnormal functional homotopy was also found in the precuneus/PCC (the posterior part of the default mode network), which plays a central role in higher-order cognitive function and neural correlates of consciousness (29). Decreased FC between these regions may be associated with deficits in self-referential processing, attentional control and working memory (30). Previous studies have demonstrated that patients with subacute and chronic stroke could have reduced FC between components in the default mode network which might correlate with cognitive dysfunction, anxiety or depression after a stroke (31, 32). Our results found homotopic FC in the precuneus/PCC stayed at a long-term lower level from stroke onset to day 180 but altered slightly with an increasing trend, which could indicate a relatively slow process of cognitive restoration.

In addition, we found brain regions with an initial increase of homotopic FC (observed in the hippocampus/amygdala and frontal pole) stayed at an elevated level for months after a stroke. These brain areas contribute to many essential aspects of cognition, such as learning, emotional events, working memory and monitoring goals of particular kinds (33–35). Actually, patterns of FC changes in cognitive areas presented in our work were consistent with previous longitudinal studies. Enhanced FC alterations associated with cognitive function could appear immediately after stroke onset and remain at high levels for weeks or months, even in patients with recovered motor function (8, 36). These changes may indicate a neural mechanism of long-term cognitive compensation for motor dysfunction. However, further studies could assess neuropsychological measures for patients with stroke to identify whether cognitive impairment is accompanied by motor deficit.

### Correlation between early homotopic FC and late motor recovery in patients with pontine infarction

Our study first demonstrates a negative correlation between early homotopic FC changes and later motor assessment (from day 14 to day 180 after stroke onset), which means an initially higher level of inter-hemispheric FC in the hippocampus/amygdala and frontal pole may predict worse motor impairment in the future. In this way, early motivation of cognitive function may reflect the severity of intrinsic brain damage in motor function. As previously mentioned, unlike FC in sensorimotor areas, FC in cognitive regions presented long-term abnormality for several weeks (even until day 180 in the frontal pole), which further confirms that cognitive function plays an essential role in motor restoration. A critical review showed clinical evidence for our findings, demonstrating how cognitive-strategy-based interventions enhance long-term motor rehabilitation after stroke (37). However, the intrinsic neural mechanism underlying cognitive compensation for motor recovery is still unclear. A previous study found positive correlations between FC in the ipsilesional M1 and contralesional middle frontal gyrus at stroke onset and motor recovery at 6 months after the stroke (24). Thus, future studies concerning this problem could focus on the functional interactions between the sensorimotor network and cognitive network.

### Possible basis of homotopic FC changes in patients with pontine infarction

In healthy people, homotopic FC alterations may be associated with gray matter changes and also reflect the integrity of the subcortical structure for interhemispheric communication (23). In patients with stroke, disruption of homotopic FC has been considered to be associated with structural impairment of fiber tracts, especially of the corticospinal tract (CST) and transcallosal fibers. A previous study has demonstrated a positive correlation between decreased FC of bilateral M1 and the extent of CST damage in patients with subacute stroke (38), while another study put forward the idea that negative correlation between enhanced interhemispheric FC and CST damage would provide evidence for regarding homotopic FC as a compensation mechanism after structural deficits in patients who had good recovery (39). Our research team have also published a series of studies discussing long-term FC and structural abnormalities in patients with pontine infarction, with some of the same data used in the current study (40–42). Together with these findings, we suggest further study could focus on longitudinal observations of correlations between homotopic FC and structural alterations, to explore possible mechanisms underlying inter-hemispheric connection in patients with stroke.

### Limitations

Our study focused on longitudinal functional homotopy alterations in patients with pontine infarction. Thus, the research sample was restricted to a narrow range of these patients with acute stroke and who achieved relatively good recovery of motor function on day 180 (participants with poor outcome were unable to complete our long-term follow up). This restriction helps to improve the homogeneity of our results, but also limits the validation of our findings for other stroke populations. We hope that further studies could be performed with a larger sample size and various subgroups of infarct loci. On the other hand, we used VMHC to quantify voxel-based homotopic FC between bilateral hemispheres, which has been proved to be a method with considerable reliability (23, 43) and has been effectively applied to patients with stroke (25, 44). However, some of the FC findings reported in this study did not reach statistical significance when multiple comparisons were corrected using permutation method. This may due to the huge number of voxels involved in the whole-brain analysis, which increases the need for a larger sample-size research. Further multi-center researches could recruit bigger sample of stroke patients to validate our findings. Also, ROI-based or network-based postprocessing methods could help to find other possible inter-hemispheric bio-markers for motor-outcome prediction.

## Conclusion

Over all, our findings demonstrated the potential utility of early homotopic FC alterations for prediction of late motor recovery in patients with stroke. In addition, we also demonstrated a long-term pattern of functional homotopy changes in patients with pontine infarction, which may contribute to further understanding of brain restoration after stroke.

## Author contributions

JL, X-NZ, and K-CL substantial contributions to the conception and design of the work. MZ, D-DR, Z-LZ, Y-XC, P-PW, and Q-FM performed data acquisition for the work. YS, Y-SW, Z-ZD, and X-NZ analyzed and discussed data for the work. YS drafted the work. YS, Y-SW, X-NZ, and JL revised the paper and finally approved the version to publish.

### Conflict of interest statement

The authors declare that the research was conducted in the absence of any commercial or financial relationships that could be construed as a potential conflict of interest.
